# How are people with mild cognitive impairment or subjective memory complaints managed in primary care? A systematic review

**DOI:** 10.1093/fampra/cmab014

**Published:** 2021-04-28

**Authors:** Brendan Hallam, Jessica Rees, Irene Petersen, Claudia Cooper, Christina Avgerinou, Kate Walters

**Affiliations:** 1 UCL Research Department of Primary Care and Population Health, London, UK; 2 Division of Psychiatry, University College London, London, UK

**Keywords:** Cognitive dysfunction, dementia, memory, primary health care, primary prevention, systematic review

## Abstract

**Background:**

Primary care is typically the first point of contact in the health care system for people raising concerns about their memory. However, there is still a lack of high-quality evidence and understanding about how primary care professionals (PCPs) currently manage people at higher risk of developing dementia.

**Objectives:**

To systematically review management strategies provided by PCPs to reduce cognitive decline in people with mild cognitive impairment and subjective memory complaints.

**Method:**

A systematic search for studies was conducted in December 2019 across five databases (EMBASE, Medline, PsycInfo, CINAHL and Web of Science). Methodological quality of included studies was independently assessed by two authors using the Mixed Methods Appraisal Tool.

**Results:**

An initial 11 719 were found, 7250 were screened and 9 studies were included in the review. Most studies were self-reported behaviour surveys. For non-pharmacological strategies, the most frequent advice PCPs provided was to increase physical activity, cognitive stimulation, diet and social stimulation. For pharmacological strategies, PCPs would most frequently not prescribe any treatment. If PCPs did prescribe, the most frequent prescriptions targeted vascular risk factors to reduce the risk of further cognitive decline.

**Conclusion:**

PCPs reported that they are much more likely to provide non-pharmacological strategies than pharmacological strategies in line with guidelines on preventing the onset of dementia. However, the quality of evidence within the included studies is low and relies on subjective self-reported behaviours. Observational research is needed to provide an accurate reflection of how people with memory problems are managed in primary care.

## Lay Summary

People will typically go to their general practitioners, also known as primary care professionals (PCPs), to raise concerns about their memory. However, there is no clear understanding of what advice or treatment PCPs provide to people with memory concerns who are at high risk of dementia. This review aims to summarize the findings from research that studied what advice or treatments PCPs would give to a person with memory concerns. Nine studies were included in the review after screening through 11 719 studies. The current review found that PCPs were more likely to provide advice rather than prescribe any drug treatment. The most common advice that PCPs provided was to increase physical activity, cognitive stimulation and social stimulation. If PCPs decided to prescribe drugs, the most common prescriptions were to improve blood flow. Improving blood flow has been linked with reducing the risk of developing dementia. However, the quality of the studies included in this review is low because many relied on PCPs answering questionnaires on their intentions to manage people with memory concerns. Therefore, future research needs to observe PCPs’ real-life practice to provide an accurate reflection of how people with memory problems are managed in primary care.

**Key Messages**

- Review of primary care professionals’ (PCPs) management of memory concerns.
- The review included a wide range of quantitative and qualitative study designs.
- Most frequent advice was to increase physical activity.
- Most common drug response was to not prescribe any treatment.
- Majority of PCPs reported strategies that followed prevention guidelines.
- Future research needs more observational studies to observe real-life practice.

## Introduction

### Background

An estimated 50 million people are expected to be living with dementia worldwide, with this projected to rise to 152 million in the next 30 years (1). Dementia is the seventh leading cause of death across the world (2) and the leading cause of death within England and Wales (3). Dementia is the only condition within the top 10 causes of death without a treatment to slow or cure its progression (3). However, it is believed that up to 40% of dementia cases could be prevented if the following risk factors were addressed: low level of education, hearing loss, traumatic brain injury, hypertension, alcohol misuse, obesity, smoking, depression, physical inactivity, social isolation, air pollution and diabetes (4).

People defined as high risk of developing dementia have been operationalized in various ways. For example, the FINGER trial (5) used the CAIDE dementia risk score, whilst other studies may use the Framingham vascular risk scores (6). However, the one indicator that often leads to consultation due to concerns about the risk of developing dementia is memory concerns (7). The term ‘memory concerns’ refers to people with subjective memory complaint (SMC) and mild cognitive impairment (MCI). SMC is defined as a form of complaint that an individual makes regarding his or her cognition, but no clear impairment is found by objective psychometric testing (8). In contrast, people with MCI do show a noticeable decline in cognition using objective testing, which is not severe enough to interfere with daily activities and be defined as dementia (9). SMC affects half of people over 65 years old (10) and MCI affects 20% of people over 65 (11). Reviews have indicated that people with SMC are twice as likely to develop dementia as individuals without SMC (12), highlighting the need for health care professionals to effectively manage people with SMC and MCI in order to reduce the risk of developing dementia.

There is low-to-moderate quality evidence that addressing hypertension (13), diabetes (14), physical activity (15), tobacco cessation (16), cognitive stimulation (17) and social isolation (17) has been demonstrated to reduce dementia risk in low-to-moderate quality evidence. Treatment addressing hearing loss (18), obesity (19) and depression (20) requires further research and has yet to demonstrate protective factors for dementia. Alcohol misuse (21) and dementia has a complex J-shaped relationship with excessive alcohol use and non-consumption being associated with greater risk than moderate consumption. However, this research addressed all risk factors individually rather than the effectiveness of a behavioural health intervention that combines strategies for multiple risk factors. Evidence from trials of time-intensive behavioural health interventions targeting the lifestyle risk factors aiming to reduce cognitive decline and onset of dementia in people with memory concerns is mixed (5,22). Further investigations of lifestyle interventions, such as Active Prevention in People at risk of dementia through Lifestyle, bEhaviour change and Technology to build REsiliEnce (APPLE-Tree) (23) and the Systematic Multi-domain Alzheimer’s Risk Reduction Trial (SMARTT) (24) are ongoing. SMARRT will recruit older adults with subjective cognitive complaints from primary care and be randomly assigned to the intervention or a health education control. The intervention will be to develop a personalized plan for risk factors hypertension, hyperglycaemia, depressive symptoms, poor sleep, polypharmacy, physical inactivity, low cognitive stimulation, social isolation, poor diet and smoking. All of these factors are associated with an increased risk of dementia and strategies addressing these issues provide the most likely approach to delay the onset of dementia. However, the efficacy of dementia prevention interventions in delaying incident dementia is still mixed and inconclusive (5,22).

Therefore, there are no current specific treatment recommendations provided by the national health governing bodies for people with memory problems (SMC and MCI) due to the lack of strong current evidence (25–27). Consequently, the current guidelines for health professionals to delay the onset of dementia is to provide generic non-pharmacological recommendations to all people in mid-life (25). This includes encouraging healthy behaviours, such as smoking cessation, increasing physical activity and reducing alcohol consumption (25).

Primary care is typically the first point of contact in the health care system for people raising concerns about their memory (28). Therefore, primary care is critically placed to play a greater role in providing preventive treatments to delay the onset of dementia in adults with memory problems (28). Despite this, dementia prevention advice or even recognition of cognitive impairment by general practitioners (GPs) is variable, often with failure to respond to memory loss symptoms (29). Godbee *et al.* (30) have recently published a preliminary conceptual model on how to implement dementia risk reduction practice in primary care, providing five implementation strategies, which were (i) identifying ‘champions’ to promote brain health to patients, (ii) conducting educational meetings, (iii) conducting local consensus discussions, (iv) altering incentive structure and (v) capturing and sharing local knowledge. However, there is still a lack of high-quality evidence and understanding about how primary care professionals (PCPs) currently manage people at higher risk of developing dementia. Therefore, this systematic review will investigate what management strategies are offered by PCPs in response to managing cognitive decline and risk of dementia in people with MCI or SMC. The review will aim to bridge the gap within the literature by exploring both pharmacological and non-pharmacological strategies recommended to people with MCI or SMC in a primary care setting.

## Methods

This review was performed in accordance with the PRISMA guidelines (31) and the protocol was registered with Prospero (ID: CRD42020170804).

### Search strategy

The systematic review was conducted on 11 December 2019 using five online bibliographic databases (EMBASE, Medline, PsycInfo, CINAHL and Web of Science). See Supplementary Figures 1–5 for full search terms used. No limits were set for time or language and authors were contacted to acquire missing or further information if needed. Forward selection and reference lists from the final included papers were manually searched to identify potentially relevant studies that may not have been captured in the literature search.

### Inclusion and exclusion

To be included, studies were required to assess pharmacological or non-pharmacological management options provided by any professional (GPs, practice nurses, pharmacists, etc.) in a primary care setting to people over 50 years old with MCI or cognitive complaint without dementia. The threshold of 50 years old was selected as acquired memory concerns are increasing and starting to be treated more seriously (32). The study could be quantitative or qualitative. Non-English language papers were accepted during initial screening. However, non-English papers were excluded during full-text screening if an English version was not be obtained. Exclusion criteria included only people with a confirmed diagnosis of dementia or healthy older adults. Intervention-based studies were excluded in order to capture real-life management practices. Additionally, interventional studies, reviews, book chapters and dissertations were also excluded. Finally, if the study focussed on diagnosis or screening rather than treatment or management, it was also excluded.

### Data extraction

Two reviewers were responsible for the screening process. The second reviewer (JR) completed a random 10% of the initial screening that was blinded to the first reviewer (BH). If interrater reliability was below 0.80 for Kappa, then another 10% of the papers would be screened by JR. However, if Kappa was above 0.80, then this would be deemed satisfactory and reviewers would progress to full-text screening. If either reviewer considered a paper potentially relevant, it was retrieved and included for the full-text screening process. Both BH and JR completed 100% of the full-text screening independently with any discrepancies resolved by a third independent reviewer.

From the studies included in the systematic review, a pre-piloted data collection form was used by BH and JR to extract the necessary data. Extracted data included: author (year), study design, setting, professionals, service users, key findings/themes, type of pharmacological recommendations and type of non-pharmacological recommendations. Study authors were contacted for any missing data or any additional data that might be deemed relevant to the review. A narrative analysis of studies was conducted using a data-driven integrated synthesis approach. Quantitative and qualitative studies were synthesized applying a transformation process known as quantitizing. Quantitizing is a method validated for mixed-method reviews whereby qualitative data are quantified. (33)

### Quality assessment

Two authors independently assessed the methodological quality of each study using the mixed-methods appraisal tool (MMAT) (34). The use of MMAT in mixed-method reviews has been validated, which then allows quality appraisal for the variety of study designs to be completed using one tool (35,36). Therefore, the MMAT was chosen to appraise both qualitative and quantitative study designs included in the current review. Similar to data extraction, the interrater reliability was deemed acceptable with Kappa equal or above 0.8, and any disagreements were discussed with a third independent reviewer.

## Results

### Study selection

**Figure 1.**
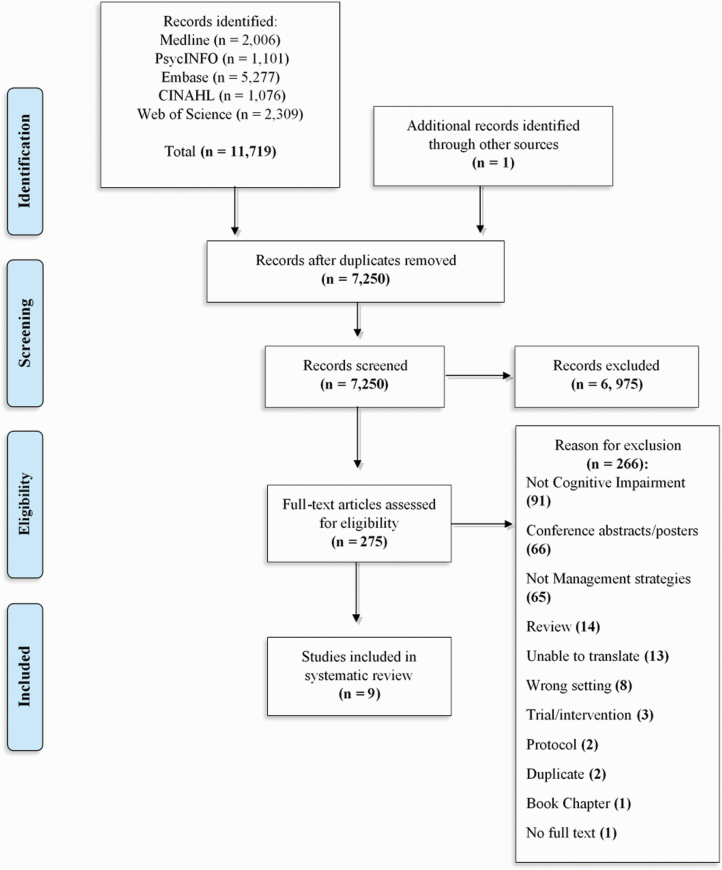
PRISMA flowchart describing the process of study selection.

The search yielded 11 719 papers. After de-duplication and the addition of one extra paper identified through other sources, 7250 title and abstracts were screened. A second independent reviewer screened 10% (*n* = 725) of the title and abstracts with a high interrater reliability (*a* = 0.89). Of 275 full-text papers retrieved, 9 were included in the final systematic review with high interrater agreement (*a* = 0.85). Figure 1 summarizes the study selection process (31).

### Characteristics and quality of included studies

We included seven quantitative studies: one descriptive naturalistic study (37), one structured interview (38) and five cross-sectional surveys (39–43) of PCPs’ self-reported management strategies. Additionally, two qualitative studies were included, one study using semi-structured interviews (44) and one case report (45). The included studies are set across seven countries (Canada, Germany, Israel, Malaysia, Spain, UK and USA), with four studies including data from the USA. A total of 2756 primary care physicians participated across eight of the included studies, with Argimon-Pallas *et al.* (37) reporting the number of primary care practices participating rather than the number of physicians. Six of the studies focussed on the management of people with MCI (37–40,44,45). Three studies focussed on SMC and memory concerns (41–43).

The methodological quality of the study designs included was of low-to-moderate quality overall. Aspects of methodology and analysis for several of the studies were unclear. None of the studies included healthy control groups to allow comparisons between managements strategies of PCPs for both cognitively healthy older adults versus people with memory problems. Argimon-Pallas *et al.* (37) was the only study using comparison groups, comparing treatments received for groups with memory problems against group with confirmed diagnosis of dementia. Another concern for each of the survey-based designs was the lack of clarity on accounting for the potential bias in response rates and investigating any difference in characteristics between responders and non-responders of the survey. The quality appraisal for all studies can be found in Supplementary Table 1 (*a* = 0.80).

### Non-pharmacological management

Two thousand one hundred and sixty-nine primary care physicians were recruited across five studies that investigated non-pharmacological management for people presenting with either memory problems (SMC or MCI). Three of the five studies were survey based, one was a case report and one was semi-structured interviews and a focus group.

### Subjective memory concern

Two studies investigated primary care physician’s non-pharmacological management intentions in response to a patient presenting with SMC (41,42). Both studies used the DocStyles survey measure. DocStyles is a web-based survey with a range of questions, including how to reduce cognitive decline in people with memory concerns using a preset list of pharmacological and non-pharmacological strategies. Across both studies, the top two recommendations were increasing physical activity and increasing cognitive stimulation (41,42). For physicians surveyed in Day *et al.*, the third most common recommendation was for the patient to improve their diet. However, in Friedman *et al.*, physicians’ third highest recommendation to patients was to increase social stimulation. A small proportion, 40 physicians (4%) from Friedman *et al.*, indicated that they would provide no advice for any treatment or strategies in preventing cognitive decline. Day *et al.* did not report if any physicians would not provide advice to patients with subjective memory concerns (please see Table 2).

### Mild cognitive impairment

For patients presenting with MCI, three studies investigated primary care physicians’ intentions to provide non-pharmacological management strategies. Werner *et al.* (40) used a survey-based measure with 11 preset pharmacological and non-pharmacological strategies, which largely overlapped with DocStyles, but had some different strategies listed. Ambigga *et al.* (45) provide a case report and vignette on how primary care physicians should manage a patient with MCI. The final study, Hochhalter *et al.* (44), conducted a qualitative study using case vignettes in focus groups and semi-structured interviews. Across all three studies (40,44,45), four recommendations were highlighted: physical activity, cognitive stimulation, social stimulation and diet. PCPs who participated in semi-structured interviews outlined the importance of recommending physical activity for a key reason ‘Vigorous daily exercise… because it improves, basically, all the vascular risks which people in this age group face’ [(44), p. 3]. The minimum requirement of what is deemed enough physical activity, or for any of the other recommendations, is not outlined across any of the studies. Hochhalter *et al.* (44) also identified a small number of PCPs who did not provide any sort of management strategies because they felt that cognitive impairment, specifically Alzheimer’s disease, is not preventable ‘Stuff like Alzheimer’s, we can’t do anything about. Either you get it, or you don’t. You can’t prevent it’ [(44), p. 3].

### Pharmacological management

Pharmacological management for people presenting with either memory problems (SMC or MCI) was investigated by all nine studies, which has been outlined above in the Characteristics and quality of included studies section (please see Table 1 for study characteristics).

**Table 1.** Study characteristics of studies included in systematic review

Author (year)	Study design	Setting	*N* of professionals [Age (*M*, SD)]	Years in practice	*N* of patients, age [*M*, SD]	Type of cognitive impairment	Pre-determined list of strategies	Key findings/themes	Type of pharmacological recommendation	Type of non-pharmacological recommendation
Quantitative										
Physician-reported management strategies										
Banjo *et al.* (43)	Survey	USA, Canada	212 primary care physicians 25–40 years old (36%) 41–59 years old (45%) 60+ years old (19%) 55% males	1–10 years (45%) 11–20 (24%) 20+ years (31%)	N/A	Case vignette—65 year old with SMC	Yes: three types of neurotropics (cognitive enhancers)	Likert scale (1–7 with 7 being highest comfort) of how comfortable physician would feel prescribing cognitive enhancers: *M* = 4.8 (SD N/A)	Modafinil Methylphenidate Sildenafil (all drugs fit the criteria for nootropics, otherwise known as cognitive enhancers)	N/A
Day *et al.* (41)	Survey	USA	493 primary care physicians 479 internist <50 years old (69%) 50+ years old (31%) 77% males	3–19 years (73%) 20+ years (27%)	N/A	Memory concerns (not specified)	Yes: 10 options (6 non-pharmacological; 3 pharmacological; 1 no treatment option)	% of physicians that would provide advice to patient on: Physical activity *n* = 892 (91.8%) Intellectual stimulation *n* = 829 (85.3%) Healthy diet *n* = 809 (83.2%) Socially activity *n* = 775 (79.7%) Limiting alcohol *n* = 626 (64.4%) Maintaining a healthy weight *n* = 511 (52.6%) Avoiding polypharmacy *n* = 434 (44.7%) Taking nutritional supplements *n* = 332 (34.2%) Taking certain new medications (not specified) *n* = 164 (16.9%)	Medication (not specified) Avoid polypharmacy Nutritional supplements (not specified)	Physical activity Social activity Diet Cognitive stimulation Limit alcohol Weight/BMI
Friedman *et al.* (42)	Survey	USA	1,000 family physicians and internist 72% males 250 nurse practitioners 13% males	14.7 years (SD = n/a) 16.4 years (SD = n/a)	N/A	Memory concerns (not specified)	Yes: 10 options (6 non-pharmacological; 3 pharmacological; 1 no treatment option)	% of physicians that would provide advice to patient on: No recommendations *n* = 40 (4%) Medication (not specified) *n* = 116 (11.6%) Take vitamins *n* = 293 (29.3%) Avoid polypharmacy *n* = 411 (41.1%) Healthy weight *n* = 457 (45.7%) Limit alcohol *n* = 591 (59.1%) Healthy diet *n* = 609 (60.9%) Socially active *n* = 667 (66.7%) Intellectual stimulation *n* = 802 (80.2%) Physically active *n* = 861 (86.1%)	Medication (not specified) Avoid polypharmacy Nutritional supplements (not specified)	Physical activity Social activity Diet Cognitive stimulation Limit alcohol Weight/BMI
Maeck *et al.* (38)	Survey (structured interview)	Germany	159 family physicians^b^ (year = 1993) 70% males 122 family physicians (year = 2001) 56% males	N/A	N/A	Case vignette—MCI	No: Open-ended questions but would categorize answers to facilitate analysis	Survey asks if family physicians would prescribe any dementia related medication to case vignette of someone with MCI who is at high risk of dementia: ^a^ = 1993 results ^b^= 2001 results Yes (any treatment) (70.4%)^a^ (43.4%)^b^ Ginkgo Biloba (34.0%)^a^ (23.0%)^b^ Pentoxiphylline (13.2%)^a^ (2.5%)^b^ Piracetam (39%)^a^ (3.3%)^b^ Nimodipine (22.0%)^a^ N/A^b^ Memantine* N/A^a^ (12.3%)^b^ Cholinesterase inhibitors N/A^a^ (8.2%)^b^ Other medication (44.7%)^a^ (0.8%)^b^	Ginkgo biloba (natural remedies) Pentoxiphylline (vascular management) Piracetam (nootropic) Nimodipine (vascular management) Memantine (anti-dementia drug) Cholinesterase inhibitors (anti-dementia drug) Medication (not specified)	N/A—not discussed
Suribhatla *et al.* (39)	Survey	UK	65 GPs % of sex not reported	N/A	N/A	Vascular cognitive impairment (VCI)	Yes: Only discussed prescription of statins and no other strategies.	*Patient with vascular cognitive impairment* 26% of GPs (16 out of 61) would prescribe statins to help manage vascular and cognitive risks *Patients at risk of VCI* 42% of GPs (27/64) felt that statins have a role in preventing VCI in at risk people	Statins (vascular management)	N/A—not discussed
Werner *et al.* (40)	Survey	Israel	197 family physicians 50.1 years old (SD = 9.2) 49% Male	21.9 years (SD = 10.4)	N/A	MCI	Yes 11 pharmacological and non-pharmacological therapies	% of family physicians (*n* = 168) preferences for treatment of MCI: Physical activities (88%) Social activities (88%) Cognitive training (88%) Engagement in support group (80%) Relaxation exercise (47%) Change in diet (44%) Psychotherapy (35%) Yoga or meditation (34%) Vitamins (34%) Pharmacological treatment (13%) Natural Medications (10%)	Pharmacological *(not specified)* Natural medication *(not specified)* Vitamins *(not specified)*	Physical activity Social activity Diet Cognitive training Relaxation/meditation therapy
Physician-observed management strategies										
Argimon-Pallas *et al.* (37)	Descriptive naturalistic study (12 months)	Spain	105 general practices N/A	N/A	921 patients reported to GP with memory concerns Male (74.9, ±6.5) Female (74.0, ±6.9)	45 diagnosed with MCI 157 cognitive impairment not dementia Other groups: 145 dementia 52 vascular dementia 73 Alzheimer’s disease 137 psychopathological disorder 25 other 126 not stated	No preset list of strategies	% of service users receiving treatment in response to cognitive impairment: *After initial visit:* Any type of treatment in response to cognitive impairment (type not specified) (76%) Nootropics (24%) Calcium antagonists (10%) *At 12 months:* Any type of treatment in response to cognitive impairment (type not specified; 76%)	Nootropics (not specified) Calcium antagonists (antihypertensive drugs)	N/A
Qualitative										
Physician-reported management strategies										
Ambigga *et al.* (45)	Case report	Malaysia	1 primary care physician	N/A	N/A	Case vignette—MCI	No preset list Provided recommendations based on evidence	Preferences for treatment: promote independence in communication and activities of daily living Mental exercise (e.g. puzzles) Healthy lifestyle including physical activity and diet Getting enough sleep Limit alcohol intake Control vascular risk factors (e.g. hypertension) Regular follow up 3–6 months	Vascular medication	Physical activity Social activity Diet Cognitive stimulation Sleep Limit alcohol
Hochhalter *et al.* (44)	Focus groups, semi-structured interviews	USA	28 primary care physicians <44 years old (53.6%) 45–64 years old (39.3%) >65 years old (7.1%) 79% male 21 advanced practice provider <44 years old (9.5%) 45–64 years old (90.5%) >65 years old (0%) 19% male	16.7 years (SD = n/a) 26.2 years (SD = n/a)	N/A	Case vignette—MCI	No preset list	Participants provided range of pharmacological and non-pharmacological strategies. However, key findings from study also outlined that some participants felt that the management options are too generic and that in some cases dementia is not preventable.	Blood pressure (vascular management) Cholesterol (vascular management) Reassessment of diabetes management (not specified)	Physical activity Social activity Diet Cognitive stimulation

*M*, mean; SD, standard deviation; N/A, not applicable (not provided).

^a^1993 results.

^b^2001 results.

### Subjective memory concern

For patients presenting with SMC, three studies investigated PCPs’ intentions for pharmacological management strategies (41–43) (please see Table 3). Both Day *et al.* (41) and Friedman *et al.* (42) used the Docstyles measure. Banjo *et al.* (43) utilized a different method by using a case vignette of a patient with memory concerns and then asking how comfortable PCPs would be prescribing cognitive enhancers. Banjo *et al.* averaged the PCPs response to how comfortable they felt prescribing a cognitive enhancer (a preset list of sildenafil, methylphenidate and modafinil) on a Likert scale with 1 being ‘Less comfortable’ and 7 being ‘More comfortable’. The PCPs felt most comfortable prescribing sildenafil. The only management response that appeared across all three studies was to provide no pharmacological response (41–43). Banjo *et al.* did not report the specific number of physicians providing advice but did report that some physicians did not provide any pharmacological response. A minimum of 1 in 5 physicians within Friedmann *et al.* and 1 in 20 physicians within Day *et al.* reported that they would not provide any pharmacological response at all. These are minimum estimates as these figures are based on adding all treatment options up, then taking that total away from the study population. However, within two studies, pharmacological response was more frequent among physicians than no treatment at all. Reducing polypharmacy was a management response to SMC being reported that just under half of physicians highlighted across two studies (41,42). Additionally, approximately a third of physicians in two studies also reported that they recommended the initiation of supplements and vitamins (41,42). However, the specific type of vitamins and supplements were not specified.

**Table 2.**
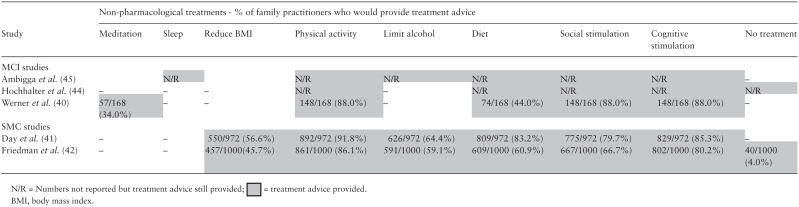
Physician behaviour of managing MCI and SMCs using a non-pharmacological response

**Table 3.**
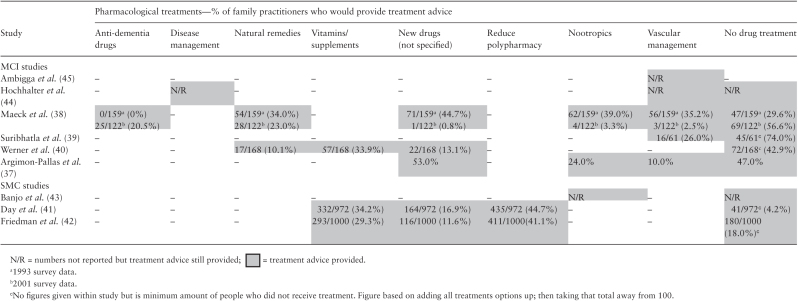
Physician behaviour of managing MCI and SMCs using a pharmacological response

### Mild cognitive impairment

Five studies investigated PCPs’ intentions and one study investigated PCPs’ observed behaviour for pharmacological management strategies for patients presenting with MCI (please see Table 3). Across four of the five studies investigating reported management strategies, physicians would not provide any pharmacological treatment in response to managing a patient with MCI. Maeck *et al.* surveyed physicians in 1993 and 2001. In 1993, just under one in three physicians reported that they would not typically provide any pharmacological treatment. In comparison to 2001, just over one in two physicians would not provide any pharmacological treatment. In a more recent survey, Werner *et al.* also indicated that just under one in two physicians reported that they would not provide any pharmacological treatment. For physicians surveyed over the last 20 years, 43% to 74% would not prescribe any form of medication (38–40,44). If physicians were to advise on the use of pharmacological treatment, vascular management appeared the most common, being highlighted across four of the five studies (38,39,44,45). Vascular management included any treatments aimed at lowering cholesterol, blood pressure and blood glucose in order to improve blood flow. One in four physicians in Suribhatla *et al.* reported that they would prescribe statins to manage vascular-related MCI. This was supported by a similar response rate of using vascular treatment management for MCI by physicians surveyed in 1993 within the Maeck *et al.* study. However, by 2001, this treatment strategy was reported by only 3 physicians out of 122 surveyed. Two studies did not report the number of physicians as one was a case report and the other was a qualitative study (44,45). Physicians within the focus groups outlined the importance of managing vascular risk factors not just for risk of conversion to dementia but also other health conditions that could occur as a result of vascular disease (44). Only one study in the review (37) investigated observed natural behaviour rather than physicians’ reported management strategies. Argimon-Pallas *et al.* (37) conducted a 12-month naturalistic descriptive study of 105 primary care centres across Spain and 202 patients who presented with cognitive impairment. Of these patients, one in four were prescribed nootropics, which are drugs aimed at enhancing cognition and can include piracetam (38), methylphenidate (43) and modafinil (43). However, the type of nootropics prescribed in Argimon-Pallas were not specified. One in 10 patients was prescribed calcium antagonists, which are primarily used for treating hypertension but can also be used for heart arrhythmia and headaches. This is a similar rate to the patients diagnosed with dementia within this study, but Argimon-Pallas *et al.* (37) did not provide analysis of any other comparator groups.

Other pharmacological strategies that PCPs reported they would use included prescription of vitamins (40), new drugs (type not specified) (38,40), review of disease management medication (such as type II diabetes) (44), natural remedies (such as Gingko Biloba) (38,40) and even anti-dementia drugs (38). In 2001, 122 PCPs in Germany (38) were given a case vignette of a patient with MCI who has an increased risk of developing dementia. At that time, 12% of PCPs (*n* = 15) would prescribe memantine and 8% (*n* = 10) would prescribe cholinesterase inhibitors to improve cognitive symptoms in people with MCI (38).

## Discussion

The review-highlighted PCPs were reporting that they were more likely to provide non-pharmacological strategies than pharmacological treatments. The three most common non-pharmacological strategies reported as being used to reduce cognitive decline and dementia risk in people with memory problems were (i) physical activity, (ii) cognitive stimulation and (iii) social stimulation (40–42,44,45). Particular types of physical activity or cognitive and social stimulation were not specified. However, current evidence suggests that not all types of physical activity are equally effective. For example, in a recent review, 4–6 months of aerobic exercise twice a week or one to three times a week combining cognitive and motor challenges (Tai Chi, dance or dumbbell training) works to improve memory and global cognitive functioning, but short-term resistance training for less than 4 months did not improve memory or cognitive functioning (46–48). While there is less evidence in the arenas of cognitive and social activities, it appears that, in these domains too, not all activity types are equally effective (17,46–48). Other key strategies that physicians reported that they used included improving diet (40–42,44,45) and reducing alcohol intake (41,42,45). However, it is important to consider that all studies on non-pharmacological management evaluated self-reported (hypothetical) behaviours and none observed actual behaviours. Additionally, three of the five studies investigating non-pharmacological strategies used preset survey lists. Therefore, these studies did not provide opportunity for physicians to outline other strategies they may implement.

For pharmacological treatment offered by PCPs for people with memory problems, the most common across eight of the nine studies was to provide no drug treatment. This appears to be in line with guidance for MCI management (49), which does not recommend any drug treatments. Additionally, treatment for memory problems is typically assessed and initiated by specialists in memory clinics or other secondary care services, which is common practice in countries in North America, Europe and Oceania (50–52). However, it is important to consider that, within two studies investigating SMCs, physicians were more frequently providing some pharmacological responses, the most common responses being vascular risk management and vitamins. As for non-pharmacological approaches, the studies did not report the specific vascular management strategies used, and not all are equally effective. For example, insulin therapy has been associated with an increased risk of developing dementia, whereas thiazolidinedione exposure is associated with protective effects and reduces the risk of dementia (14). Some evidence has indicated that all classes of antihypertensives may have protective effects for dementia with minimal difference in effect between classes (53). For vitamin or supplement management, low levels of vitamin D (54) or B vitamins (55) (B6, folate and B12) are typically associated with increased risk of dementia and are specific vitamin deficiencies that PCPs could address with minimal adverse effects.

Despite mixed evidence, the World Health Organization (48) has set out a list of strategies for managing people at high risk of developing dementia that are appropriate for PCPs across the world to deliver. This review has demonstrated that most PCPs’ reported management strategies are adhering to most of the generic recommendations outlined in the WHO report. However, within the included studies, there were some important omissions of management strategies that PCPs did not report as offering to people with memory problems. Depression, smoking and hearing loss are associated with an increased risk of developing dementia, yet no study or PCPs acknowledged this as an important strategy. Additionally, it is important to note that most of the included studies are reported strategies from PCPs and, therefore, may not accurately portray behaviours in observed practice. The only study to use a descriptive naturalistic design, which was conducted in 2007, demonstrated that neurotropics (cognitive enhancers) were being prescribed more than is being recommended (37). This is perhaps surprising given the lack of evidence to suggest the effectiveness of neurotropics or acetylcholinesterase inhibitors in people with MCI and SMC (56,57). In particular, acetylcholinesterase inhibitor prescription in MCI should not be recommended due to many safety issues and minimal improvement in cognition (57).

Primary care is in an optimal position to not only first identify people with memory concerns and problems but also to coordinate the management of risk after the patient is screened as having SMC or MCI. Therefore, it is important that PCPs advise people with memory problems on the modifiable health and lifestyle factors associated with dementia, such as hypertension, depression, hearing loss and the other nine factors identified in the Lancet commission (4). By informing patients of these strategies, people with memory problems could reduce the risk of further cognitive decline or delay the onset of dementia.

### Limitations

There are some limitations to consider when interpreting the findings of this study. Due to heterogeneity in location, population and methods across different studies, we did not pool data across the studies for a meta-analysis. We employed inclusive eligibility criteria in terms of study design, which allowed survey-based studies, qualitative interviews and observational studies to be synthesized together. The included studies were conducted across a range of countries, with different guidelines for practice, which may have impacted on the strategies reported by the PCPs. A major limitation of all studies was that control groups were not used to compare how treatment for an older patient at high risk of developing dementia might differ from an older patient with no memory problems. Therefore, the percentage of people with memory problems who receive non-pharmacological recommendations, such as diet, physical activity and social stimulation, may be the same percentage of older people who would anyway receive non-pharmacological recommendations as part of general health promotion advice or to treat other conditions. The lack of description, especially for pharmacological treatments, made it difficult to know the specific types of drugs used. For example, Argimon-Pallas *et al.* (37) used the term nootropics, which is a generic term for substances that aim to improve cognition, and can range from caffeine to Ritalin.

Other limitations in relation to the methodology of the current review are only selecting English language studies. The current review did not have the capacity or resources to translate non-English articles, which could introduce bias if potential key data from non-English articles are missed. Additionally, due to limited resources, the review also prioritized peer-reviewed articles to maintain the scientific standard of the literature included in the review and excluded grey literature.

### Future research

Though self-report measures may provide some correspondence to observed behaviour, there are still large discrepancies between self-reported attitudes and actual observed behaviours (58,59). To gain a more accurate reflection of primary care current management strategies for people with MCI or SMC, high-quality longitudinal observational studies are needed. Observational studies can provide an insight into if people with memory problems are actively being managed differently than people who are cognitively healthy. Future research should monitor both pharmacological and non-pharmacological dementia prevention strategies offered by primary care. Research should also capture the specific types of management strategies offered, such as aerobic exercise or weight training for physical activity.

## Conclusion

The current review highlighted that when people are presenting with memory problems, primary care physicians will suggest that the patient can mitigate cognitive decline by improving physical activity, cognitive stimulation, social stimulation and diet. Addressing hearing loss, smoking and depression were not mentioned as strategies. For MCI, most physicians report that they will not intend to prescribe any pharmacological treatments; but if they did, it would most likely be to manage vascular risk factors. For SMC, there were physicians across all three studies that provided no pharmacological treatment at all. However, in two studies, physicians were more likely to reduce polypharmacy and increase vitamins than to provide no treatment at all. Most studies were surveys of subjective self-reported behaviours and there is a lack of strong evidence to accurately answer what are the current treatment responses for people with memory problems provided by PCPs. Future research using observational study designs is needed to obtain a more accurate reflection of actual current practice rather than reported practice. By understanding current practices, research can optimize the management of cognitive decline and dementia prevention in primary care.

## Supplementary Material

cmab014_suppl_Supplementary_Material

## Declaration

Funding: Brendan Hallam is a PhD student funded by the Economic & Social Research Council’s London (UBEL) Doctoral Training Partnership, embedded within the APPLE-TREE programme (Project reference: ES/S010408/1).

Ethics approval: none.
