# Supplementary material for: How are people with mild cognitive impairment or subjective memory complaints managed in primary care? A systematic review
Source: Fam Pract. 2021 Apr 28;38(5):669–83. doi: 10.1093/fampra/cmab014 (PMC8604277; doi:10.1093/fampra/cmab014)
Supplement: cmab014_suppl_Supplementary_Material [file cmab014_suppl_Supplementary_Material.docx]

**SUPPLEMENTARY MATERIAL**

*Supplementary figure 1: Full search terms used for EMBASE*

**EMBASE SEARCH**

**11.12.19**

**Key Concept 1: Mild cognitive impairment**

# Searches

1 MCI.mp.

2 mild cognitive impairment.mp.

3 subjective memory impairment.mp.

4 subjective cognitive impairment.mp.

5 mild neurocognitive disorder.mp.

6 Mncd.mp.

7 cognit* impair*.mp.

8 (subjective adj5 (memory complaint* or memory impair* or memory chang* or memory perform* or memory declin* or memory problem*)).mp.

9 (perceiv* adj5 (memory complaint* or memory impair* or memory chang* or memory perform* or memory declin* or memory problem*)).mp.

10 (perception* adj5 (memory complaint* or memory impair* or memory chang* or memory perform* or memory declin* or memory problem*)).mp.

11 (measurable adj5 (memory complaint* or memory impair* or memory chang* or memory perform* or memory declin* or memory problem*)).mp.

12 cognitiv* declin*

13 cognitive dysfunction*

14 cognitive deficit.mp.

15 (risk adj5 dementia).mp.

16 "disorders of higher cerebral function"/

17 mild cognitive impairment/

18 cognitive defect/

19 memory disorder/

20 or/1-19

**Key Concept 2: Primary Care**

# Searches

21 primary care.mp.

22 GP.mp.

23 general practi*.mp.

24 family practi*.mp.

25 family physician.mp.

26 community nurse.mp.

27 community practi*.mp.

28 family nurse.mp.

29 community health nurse.mp.

30 exp General Practice/

31 general practitioner/

32 family nurse practitioners/

33 community care/ or community health nursing/

34 visiting nursing service/

35 primary nursing/

36 exp primary health care/

37 OR/21-36

**COMBINING two concepts**

37 20 AND 37

**= 5,227 results**

*Supplementary figure 2: Full search terms used for Medline*

**Medline SEARCH (11.12.19)**

**Ovid MEDLINE(R) and Epub Ahead of Print, In-Process & Other Non-Indexed Citations and Daily 1946 to November 08, 2019**

**Key concept 1: MCI**

# Searches

1 MCI.mp.

2 mild cognitive impairment.mp.

3 subjective memory impairment.mp.

4 subjective cognitive impairment.mp.

5 mild neurocognitive disorder.mp.

6 Mncd.mp.

7 cognit* impair*.mp.

8 cognitiv* declin*.mp

9 cognitive dysfunction*.mp.

10 cognitive deficit.mp.

11 (subjective adj5 (memory complaint* or memory impair* or memory chang* or memory perform* or memory declin* or memory problem*)).mp.

12 (perceiv* adj5 (memory complaint* or memory impair* or memory chang* or memory perform* or memory declin* or memory problem*)).mp.

13 (perception* adj5 (memory complaint* or memory impair* or memory chang* or memory perform* or memory declin* or memory problem*)).mp.

14 (measurable adj5 (memory complaint* or memory impair* or memory chang* or memory perform* or memory declin* or memory problem*)).mp.

15 (risk adj5 dementia).mp.

16 Memory Disorders/

17 Cognition Disorders/

18 Cognitive Dysfunction/

19 or/1-18

**Key concept 2: Primary Care**

# Searches

20 primary care.mp.

21 primary health care.mp.

22 GP.mp.

23 general practi*.mp.

24 family practi*.mp.

25 family physician.mp.

26 community nurse.mp.

27 community practi*.mp.

28 family nurse.mp.

29 community health nurse.mp.

30 primary health care/

31 exp General Practice/

32 general practitioners/ or physicians, family/ or physicians, primary care/

33 family nurse practitioners/ or nurses, community health/ or occupational health physicians/

**Combining**

34 or/20-33

35 19 and 34

**= 2,006 results**

*Supplementary figure 3: Full search terms used for PsycINFO*

**PsycINFO SEARCH**

**11.12.19**

**Key concept 1: MCI**

# Searches

1 MCI.mp.

2 mild cognitive impairment.mp.

3 subjective memory impairment.mp.

4 subjective cognitive impairment.mp.

5 mild neurocognitive disorder.mp.

6 Mncd.mp.

7 cognit* impair*.mp.

8 (subjective adj5 (memory complaint* or memory impair* or memory chang* or memory perform* or memory declin* or memory problem*)).mp.

9 (perceiv* adj5 (memory complaint* or memory impair* or memory chang* or memory perform* or memory declin* or memory problem*)).mp.

10 (perception* adj5 (memory complaint* or memory impair* or memory chang* or memory perform* or memory declin* or memory problem*)).mp.

11 (measurable adj5 (memory complaint* or memory impair* or memory chang* or memory perform* or memory declin* or memory problem*)).mp.

12 cognitiv* declin*.mp.

13 cognitive dysfunction*.mp.

14 cognitive deficit.mp.

15 (risk adj5 dementia).mp.

16 Cognitive impairment/

17 Memory disorders/

18 Cognitive ability/ or exp cognitive ageing/

19 OR/1-18

**Key concept 2: Primary Care**

# Searches

20 primary care.mp.

21 primary health care.mp.

22 GP.mp.

23 general practi*.mp.

24 family practi*.mp.

25 family physician.mp.

26 community nurse.mp.

27 community practi*.mp.

28 family nurse.mp.

29 community health nurse.mp.

30 exp primary health care

31 exp general practitioners/

32 exp family physicians/

33 or/20-32

**Combining**

34 19 and 33

**= 1,101 results**

*Supplementary figure 4: Full search terms used for CINAHL*

**CINAHL SEARCH**

**11.12.19**

**Key concept 1: MCI**

# Searches

1 MCI.mp.

2 “mild cognitive impairment”.mp.

3 “subjective memory impairment”.mp.

4 “subjective cognitive impairment”.mp.

5 “mild neurocognitive disorder”.mp.

6 Mncd.mp.

7 cognit* impair*.mp.

8 (subjective N5 (memory complaint* or memory impair* or memory chang* or memory perform* or memory declin* or memory problem*)).mp.

9 (perceiv* N5 (memory complaint* or memory impair* or memory chang* or memory perform* or memory declin* or memory problem*)).mp.

10 (perception* N5 (memory complaint* or memory impair* or memory chang* or memory perform* or memory declin* or memory problem*)).mp.

11 (measurable N5 (memory complaint* or memory impair* or memory chang* or memory perform* or memory declin* or memory problem*)).mp.

12 “cognitiv* declin*”.mp.

13 “cognitive dysfunction*.mp.

14 “cognitive deficit”.mp.

15 (risk N5 dementia).mp.

16 cognition disorders/

17 memory impairment (saba CCC)/

18 Impaired memory (NANDA)/

19 OR/1-18

**Key concept 2: Primary Care**

# Searches

20 “primary care”.mp.

21 “primary health care”.mp.

22 GP.mp.

23 “general practi*”.mp.

24 “family practi*”.mp.

25 “family physician”.mp.

26 “community nurse”.mp.

27 “community practi*”.mp.

28 “family nurse”.mp.

29 “community health nurse”.mp.

30 primary health care/

31 physicians, family/

32 Community health nursing/

33 or/20-32

**Combining**

33 19 and 33

**= 1,076 results**

*Supplementary figure 5: Full search terms used for Web of Science*

**Web of Science SEARCH**

**02.12.19**

**Key concept 1: MCI**

# Searches

1 MCI.mp.

2 “mild cognitive impairment”.mp.

3 “subjective memory impairment”.mp.

4 “subjective cognitive impairment”.mp.

5 “mild neurocognitive disorder”.mp.

6 Mncd.mp.

7 “cognit* impair*”.mp.

8 ((subjective NEAR/5 memory complaint*) or (subjective NEAR/5 memory impair*) or (subjective NEAR/5 memory chang*) or (subjective NEAR/5 memory perform*) or (subjective NEAR/5 memory declin*) or (subjective NEAR/5 memory problem*)).mp.

9 ((perceiv* NEAR/5 memory complaint*) or (perceiv* NEAR/5 memory impair*) or (perceiv* NEAR/5 memory chang*) or (perceiv* NEAR/5 memory perform*) or (perceiv* NEAR/5 memory declin*) or (perceiv* NEAR/5 memory problem*)).mp.

10 ((perception* NEAR/5 memory complaint*) or (perception* NEAR/5 memory impair*) or (perception* NEAR/5 memory chang*) or (perception* NEAR/5 memory perform*) or (perception* NEAR/5 memory declin*) or (perception* NEAR/5 memory problem*)).mp.

11 ((measurable NEAR/5 memory complaint*) or (measurable NEAR/5 memory impair*) or (measurable NEAR/5 memory chang*) or (measurable NEAR/5 memory perform*) or (measurable NEAR/5 memory declin*) or (measurable NEAR/5 memory problem*)).mp.

12 “cognitiv* declin*”.mp.

13 “cognitive dysfunction*”.mp.

14 “cognitive deficit”.mp.

15 cognition disorders.mp.

16 (risk NEAR/5 dementia).mp.

17 OR/1-16

**Key concept 2: Primary Care**

# Searches

18 “primary care”.mp.

19 “primary health care”.mp.

20 GP.mp.

21 “general practi*”.mp.

22 “family practi*”.mp.

23 “family physician”.mp.

24 “community nurse”.mp.

25 “community practi*”.mp.

26 “family nurse”.mp.

27 “community health nurse”.mp.

28 or/18-27

**Combining**

28 17 and 28

**= 2,309 results**

*Supplementary Table 1: Quality appraisal of studies included in the systematic review using the Mixed Methods Appraisal Tool (MMAT)*

|  | **SCREENING QUESTIONS** | | **1. QUALITATIVE STUDIES** | | | | | **COMMENTS** |  |
| --- | --- | --- | --- | --- | --- | --- | --- | --- | --- |
| Citation | S1. Are there clear research questions? | S2. Do the collected data allow to address the research questions? | 1.1. Is the qualitative approach appropriate to answer the research question? | 1.2. Are the qualitative data collection methods adequate to address the research question? | 1.3. Are the findings adequately derived from the data? | 1.4. Is the interpretation of results sufficiently substantiated by data? | 1.5. Is there coherence between qualitative data sources, collection, analysis and interpretation? |  |  |
| Ambigga et al., (2011) | Yes | No | Yes | Can't tell | No | Yes | Yes | Case study. All recommendations made are based on literature and guidelines previously identified and paper looks to provide as overview of diagnosis and management of MCI by primary care professional. No information on how data that formed case example was collected. |  |
|  |  |  |  |  |  |  |  |  |  |
| Hochhalter et al., (2012) | Yes | Yes | Yes | Yes | Yes | Yes | Yes | Is a qualitative study consisting of focus groups and semi-structured interviews. Methodology to collect data appears to be of High quality. However, there is no clear method for analysis of data. Does not outline if intention is to use thematic analysis or ground theory for example. Could improve results by grouping into themes. |  |
|  | **SCREENING QUESTIONS** | | **2. RANDOMIZED CONTROLLED TRIALS** | | | | | **COMMENTS** |  |
| Citation | S1. Are there clear research questions? | S2. Do the collected data allow to address the research questions? | 2.1. Is randomization appropriately performed? | 2.2. Are the groups comparable at baseline? | 2.3. Are there complete outcome data? | 2.4. Are outcome assessors blinded to the intervention provided? | 2.5 Did the participants adhere to the assigned intervention? |  |  |
| None |  |  |  |  |  |  |  |  |  |
|  | **SCREENING QUESTIONS** | | **3. NON-RANDOMIZED STUDIES** | | | | | **COMMENTS** |  |
| Citation | S1. Are there clear research questions? | S2. Do the collected data allow to address the research questions? | 3.1. Are the participants representative of the target population? | 3.2. Are measurements appropriate regarding both the outcome and intervention (or exposure)? | 3.3. Are there complete outcome data? | 3.4. Are the confounders accounted for in the design and analysis? | 3.5. During the study period, is the intervention administered (or exposure occurred) as intended? |  |  |
| None |  |  |  |  |  |  |  |  |  |
|  | **SCREENING QUESTIONS** | | **4. QUANTITATIVE DESCRIPTIVE STUDIES** | | | | | **COMMENTS** |  |
| Citation | S1. Are there clear research questions? | S2. Do the collected data allow to address the research questions? | 4.1. Is the sampling strategy relevant to address the research question? | 4.2. Is the sample representative of the target population? | 4.3. Are the measurements appropriate? | 4.4. Is the risk of nonresponse bias low? | 4.5. Is the statistical analysis appropriate to answer the research question? |  |  |
| Argimon Pallas et al., (2007) | Yes | Yes | Yes | Can’t tell | Can’t tell | No | No | Descriptive naturalistic study monitoring observed behaviour. Treatment response directly after consultation and then again in 6 months time. No control or comparison group on healthy older adults provided. Lack of detail on specific types of medication prescribed. Did not provide enough detail on difference between treatment recommendations for people with MCI compared to SMC or Dementia. How data was collected was unclear. |  |
| Banjo et al (2010) | Yes | No | No | Can’t tell | Can’t tell | Can’t tell | No | Case-vignette based Survey to understand reported management behaviours. Participants were recruited by mailing out letters and see who responded. Cannot tell on risk of bias as no information on number of people survey has been sent to is provided. Reported management behaviour rather than actual observed behaviour of primary care physicians. Unclear if self-report measure was blinded. Analysis did not outline n of people who did or did not feel comfortable prescribing drug. Used a population average of comfort level. |  |
|  |  |  |  |  |  |  |  |  |  |
| Day et al (2012) | Yes | Yes | Yes | Can’t tell | Can’t tell | No | Yes | Quantitative Survey to understand reported management strategies. Randomly selected participants from 135,000 physicians until the quote of 1,000 participants was reached. 7,205 physicians were recruited but the final number of participants who completed all questions was 972 participants. No mention of comparing differences between physicians who completed the survey and those that did not. Additionally, no mention of blinding self-report measure which can increase bias. Pre-set lists of management strategies with some omissions of strategies for known dementia risk factors such as depression and hearing loss. |  |
|  |  |  |  |  |  |  |  |  |  |
| Friedman et al., (2013) | Yes | Yes | Yes | Can’t tell | Can’t tell | No | Yes | Same methodology for survey as Day (2012), except response rate was slightly higher at 45%. Also conducted stratified randomly selection to make sure included participants as whole represented age, sex and region of all physicians registered in the American Medical Association (AMA). Unclear if responders’ vs non-responders were compared. Pre-set lists of management strategies with some omissions of strategies for known dementia risk factors such as depression and hearing loss. |  |
|  |  |  |  |  |  |  |  |  |  |
| Maeck et al., (2008) | Yes | Yes | Can’t tell | Can’t tell | Can’t tell | No | Yes | Case vignette structured interview survey. Descriptive study to outline reported management strategies of PCPs. Data was collected on Two time points, 1992 and 2001. Exploratory analysis found that age, sex, location, qualification had no impact on readiness to participate in study. No mention of blinding self-report measure which can increase bias. Pre-set list of specific pharmacological strategies |  |
|  |  |  |  |  |  |  |  |  |  |
| Suribhatla, Dennis & Potter, (2005) | Yes | Yes | Can’t tell | Can’t tell | Can’t tell | No | Yes | Postal survey with 51% response rate from GPs. Unclear as to whether sampling method was opportunistic or random. No results on participant demographics but did outline that responders and non-responders were similar in age and sex since qualification. No mention of blinding self-report measure which can increase bias. Restricted to just prescription of statins and no discussion of other treatments. |  |
|  |  |  |  |  |  |  |  |  |  |
| Werner, Heinik & Kitai (2013) | Yes | Yes | No | Can’t tell | Can’t tell | Unclear | Yes | Descriptive survey of family physicians. Convenience sample. Limited information regarding response rate and whether demographics are representative of intended population. no mention of blinding self-report measure which can increase bias. Pre-set list of strategies |  |
|  | **SCREENING QUESTIONS** | | **5. MIXED METHODS STUDIES** | | | | | **COMMENTS** |  |
| Citation | S1. Are there clear research questions? | S2. Do the collected data allow to address the research questions? | 5.1. Is there an adequate rationale for using a mixed methods design to address the research question? | 5.2. Are the different components of the study effectively integrated to answer the research question? | 5.3. Are the outputs of the integration of qualitative and quantitative components adequately interpreted? | 5.4. Are divergences and inconsistencies between quantitative and qualitative results adequately addressed? | 5.5. Do the different components of the study adhere to the quality criteria of each tradition of the methods involved? |  |  |
| None |  |  |  |  |  |  |  |  |  |
